# Acceptance of ambulatory blood pressure monitoring in a semi-rural population in South Africa

**DOI:** 10.4102/hsag.v25i0.1336

**Published:** 2020-06-08

**Authors:** Farisai Chiwanza, Yoland Irwin, Ros Dowse

**Affiliations:** 1 Department of Pharmacy Practice, Faculty of Pharmacy, Rhodes University, Grahamstown, South Africa

**Keywords:** ambulatory blood pressure monitoring, acceptance, hypertension, adverse effects, primary health care

## Abstract

**Background:**

Ambulatory blood pressure monitoring is a valuable tool that helps in providing an insight into the diagnosis and management of hypertension; however, no evidence exists of its acceptance in the diverse South African population.

**Aim:**

We assessed the acceptance of an ambulatory blood pressure monitor in patients attending public sector primary health care (PHC) clinics.

**Setting:**

Five PHC clinics in the Makana subdistrict in the Eastern Cape.

**Method:**

A cross-sectional study was conducted with 70 hypertensive patients. Eligible patients were between 40 and 75 years old, taking either enalapril and hydrochlorothiazide or enalapril, hydrochlorothiazide and amlodipine. Socio-demographic, clinical and acceptance data were collected. The monitor cuff remained in place for 24 h. Acceptance was assessed after the monitor was removed. An overall acceptance score was generated to classify acceptance as either good or poor.

**Results:**

The mean years of schooling was 5.9 years, with 22 reporting no school attendance. Generally, acceptance was good, with 70% of the population rating the technique as ‘acceptable’ (acceptance score of > 23/30). Most participants reported minimal discomfort with only 13.3% reporting that it hindered normal daily activities. Night readings interrupted sleep in 43%, with extreme sleep disturbance (≥ 3 awakenings) reported in just over half the patients. Increased years of schooling was the only variable associated with acceptance score (*r* = −0.243, *p* = 0.042).

**Conclusion:**

Ambulatory blood pressure monitoring was generally well-accepted, with few adverse effects being reported. Use of this technique at PHC facilities could reduce the incidence of misdiagnosis and uncontrolled hypertension.

## Introduction

South Africa faces multiple health challenges, characterised by a quadruple burden of diseases with the burden fast shifting towards chronic non-communicable diseases (NCDS) (Bygbjerg 2012). Hypertension is a major contributor to cardiovascular complications in South Africa. The South African Health Review 2018 reported a national prevalence of 28.2% (both sexes) for these complications, and the numbers continue to rise with the increase in the prevalence of the condition in younger patient groups over the years (Gray & Vawda 2018). Further exacerbating the epidemic is rapid urbanisation combined with the globalisation of unhealthy lifestyles, which, in combination with low levels of awareness of the condition, result in hypertension being a major health problem (Gómez-Olivé et al. 2017). In response to the rapidly escalating epidemic, South Africa identified hypertension as a priority disease, which is one of the most commonly treated conditions at primary health care (PHC) facilities (Day et al. 2014; National Department of Health 1998). However, in spite of the implementation of national clinical guidelines for the diagnosis and management of hypertension at the PHC level, the condition continues to be under-diagnosed, resulting in a number of patients remaining untreated (Addo, Smeeth & Leon 2007).

Ambulatory blood pressure monitoring (ABPM) is often referred to as the ‘gold standard’ in blood pressure (BP) assessments, as it provides an insight into fluctuations over a 24-h period. The technique can be used in the initial diagnosis of hypertension, identification of the different types of hypertension (white coat, masked or nocturnal hypertension and hypertension during pregnancy) and any other discrepancies in BP (Chavanu, Merkel & Quan 2008; Mancia & Parati 2000; O’Brien 2003). Ambulatory BP monitoring also plays an important role in identifying uncontrolled hypertension and may assist in the prevention of target organ damage and possible risk of cardiovascular complications, detection of suspected sleep apnoea, as well as detection of orthostatic hypotension where this cannot be demonstrated by office BP monitoring (Rosendorff et al. 2007; Seedat, Rayner & Veriava 2014; Steyn et al. 2013).

Successful implementation and utilisation of any health technology or intervention is largely dependent on the acceptance of the technique by the target population, with acceptance being influenced by multiple factors ranging from characteristics and perceptions of the individual, to influences of societal perceptions, norms and values (Calnan, Montaner & Horne 2005; Cooper, Hill & Powe 2002; Lai et al. 2010; Peek et al. 2014). However, the majority of research on ABPM has been conducted in high-income countries, with information on its use in low- and middle-income countries (LMIC) being scant (Pickering, Shimbo & Haas 2006; Seedat 2000) and the acceptance and tolerability of this technique remaining relatively unknown.

This study sought to assess the acceptance of the Oscar 2™ ambulatory blood pressure monitor in public-sector outpatients with hypertension and to identify the determinants influencing the acceptance of ABPM in a South African semi-rural population.

## Research method and design

### Study design and setting

The study was a cross-sectional, quantitative population study. Management of hypertension in South Africa in patients relying on public sector facilities mostly occurs at PHC clinics in close proximity to their homes (Day et al. 2014). In the Eastern Cape (where the study was conducted), the incidence of hypertension is 23.7 per 1000, which is higher than the national incidence of 19.6 per 1000 (Day, Gray & Ndlovu 2018). Approximately, 13.2% of the local population has no source of income, and the majority of the population of Grahamstown relies on healthcare facilities in the public sector (Statistics South Africa 2012; ed. Broumels 2014).

### Study population and sampling strategy

The development process of the study leading to its initial stages relied substantially on the involvement of stakeholders. The district pharmacist from the Makana subdistrict health office was consulted, and a representative from that office then communicated with the nurses in charge of each clinic to initiate the implementation of the recruiting phase of the study.

The study sample comprised 70 hypertensive patients who were receiving medicines for hypertension at five public-sector PHC clinics in Grahamstown. To be eligible for the study, the participant had to be a known hypertensive (defined according to World Health Organization (WHO) as originally having a systolic reading ≥ 140 mm Hg and/or diastolic reading ≥ 90 mm Hg before starting treatment) (WHO 2013); be between the ages of 40 and 75 years and be taking either an enalapril or hydrochlorothiazide (HCTZ) or an enalapril, HCTZ and amlodipine combination for B.P. control. Individuals were excluded if they showed signs of, or a history of, cardiovascular complications and/or major target organ damage (history of stroke, congestive heart failure and clinically significant hepatic or renal diseases), persistent atrial fibrillation or other forms of arrhythmia. Persons who were smokers, pregnant or lactating females, as well as individuals with an arm circumference > 50 cm were also excluded from the study, as these conditions potentially affect readings obtained using ABPM (Viera, Lingley & Hinderliter 2011).

A screening questionnaire was developed to assist clinic personnel with the identification of potential participants. Initial screening and recruitment were conducted using two methods; firstly, the screening questionnaire was left with the pharmacist assistant or nurses at each clinic to help identify potential participants on the required medicine regimen, and secondly, convenience sampling was then employed at the clinics by the primary researcher assisted by an interpreter using the screening questionnaire.

### Data collection

The main questionnaire was designed to collect data on demographics, medical history, lifestyle, health literacy and acceptance of wearing the monitor. The Multidimensional Screener of Functional Health Literacy (MSFHL), developed in Brazil, was modified and utilised to assess health literacy in the target population.

Questions for assessing acceptance were adapted from the literature (Ernst & Bergus 2003; Viera et al. 2011; Walker et al. 2004) and modified to ensure their appropriateness for this study population. Responses for assessing acceptance were in the form of a Likert scale. In spite of Likert scales commonly ranging between 5 and 10 points, problems in interpreting the scale values have previously been noted in study populations where the majority are either of low or limited literacy. The original 10-point Likert scale was modified to a three-point Likert scale, which made the more restricted choice range easier for the participants to interpret (D’Alonzo 2011; Dowse 2016). Williams and Swanson (2001) showed that the latter is as effective as the former in obtaining the required information, with participants preferring the three-point scale, as it simplified the choice process. Additional dichotomous (yes or no) questions explored positive and negative outcomes of wearing the monitor.

All participants received invitation letters (translated into isiXhosa) providing detailed information on the study and signed a consent form during the screening stage of the study. Both documents were translated into isiXhosa by a subject specialist from the African Languages Studies, School of Languages, at Rhodes University, and back-translated by a different individual.

After the screening process, patients were visited at home on a scheduled date by the primary researcher (F.C.) for an interview and to collect data on medical history from the participant’s health passport (an outpatient booklet containing medical records, which is retained by the patient). Three office BP readings were taken using an electronic Tensoval™ monitor, and a follow-up date for the 24-h ABPM session was agreed upon.

At the follow-up session, three office BP readings were taken prior to fitting of the monitor cuff. The Oscar 2™ Ambulatory Blood Pressure Monitor cuff was placed on the non-dominant arm and remained in place both at work and at home for 24 h. The monitor was programmed to record BP readings every 30 min during the day (06:00 to 22:00 h) and every 60 min at night (22:00 to 06:00 h).

For the duration of the 24-h ABPM period, participants were encouraged to continuously wear the cuff. They were instructed to maintain their usual daily routine but refrain from bathing or engaging in any vigorous activity (e.g. jogging) and were asked to remove any tight-fitting jewellery such as a watch or ring for the duration of the ABPM. Participants were also instructed to keep the arm with the attached cuff motionless (standing or sitting) at the time when the reading was taken. In the event of the monitor failing to take a reading the first time around, it was emphasised that a repeat measurement would be attempted, and the participant should again keep the arm motionless. At the follow-up visit the following morning, the cuff was removed, and participants were encouraged to ask questions in relation to their BP and the ABPM technique. In recognition of their time and contribution to the study, on completion, the participants were offered a R100 (~$7) voucher from a local supermarket.

### Data analysis

Outcome data were summarised using descriptive statistics and frequency distributions. Likert-scaled question responses and other continuous questions were expressed as means with standard deviations, and responses to categorical questions as percentages. Comparisons for statistical significance and associations between both explanatory (e.g. age, gender, comorbidities and medicines taken) and response variables (e.g. systolic BP, diastolic BP) with acceptance outcomes were investigated by means of variance tests (e.g. ANOVA, *t*-tests) and correlation tests (Pearson correlation test). The IBM SPSS statistics v24 statistical analysis computer package was utilised for the analysis. Statistical significance was set at *p* < 0.05 for all tests.

Acceptance of the ABPM technique was reported via the use of an acceptance score generated by summating responses from the 15 opinion-based Likert scale questions. Each question was worth two points, with the maximum total score being 30. Scores lower than the 25th percentile (value = 23) were considered to reflect poor acceptance.

### Ethical consideration

Ethical approval for this study was obtained from Rhodes University Pharmacy Ethics Committee (PHARM 2015-7) and the approval to work in the public clinics was obtained from the National Department of Health, Eastern Cape (EC_2015RP7_92).

## Results

### Demographics, clinical characteristics and health literacy

Seventy participants were enrolled in the study. Table 1 presents a profile of the study population, in relation to their demographic and clinical characteristics. Three quarters of the participants were female (75.7%) and all but one participant was black African. Participant ages ranged from 45 to 75 years, with a mean age of 58.9 ± 9.1 years. Almost two-thirds had only some primary school education (≤ 7 years) and the majority were unemployed (77.1%). Health literacy in this population was low, with a mean health literacy score of 2.4 out of a maximum of 10, and the majority (73%) were categorised as having inadequate health literacy.

**TABLE 1:** Participant demographics and clinical characteristics (*n* = 70).

Characteristic	*n*	%	Mean	SD
**Gender**				
Male	17	24.3	-	-
Female	53	75.7	-	-
Age (yrs)	-	-	58.9	9.1
**Education**				
No schooling	16	22.9	-	-
Primary	28	40.0	-	-
High school or tertiary	26	37.1	-	-
Years of schooling	-	-	5.9	4.8
**Health literacy category (MSFHL)**				
Inadequate health literacy (0–3)	51	73.0	-	-
Marginal health literacy (4–5)	3	4.1	-	-
Adequate health literacy (≥ 6)	16	22.9	-	-
**Employment**				
Yes	16	22.9	-	-
No	54	77.1	-	-
**Weight**	-	-	80.9	22.8
BMI (kg/m^2^)	-	-	30.9	8.1
Normal (< 25)	14	20.0	-	-
Overweight (25–29.9)	22	31.4	-	-
Obese (≥ 30)	34	48.6	-	-
**Arm circumference**				
< 35	46	65.7	-	-
≥ 35	24	34.3	-	-
**Medicine regimen**				
HCTZ or enalapril	38	54.3	-	-
HCTZ or enalapril or amlodipine	32	45.7	-	-
**Number of comorbidities**				
0	29	41.4	-	-
1	35	50.0	-	-
2	5	7.2	-	-
≥ 3	1	1.4	-	-
**Blood pressure category**				
Normal	40	57.1	-	-
Mild	22	31.4	-	-
Moderate	6	8.6	-	-
Severe	2	2.9	-	-

MSFHL, Multidimensional Screener of Functional Health Literacy; HCTZ, hydrochlorothiazide; BMI, body mass index.

All participants had been diagnosed with hypertension, with just over half taking the enalapril or HCTZ regimen. The average weight was 80.9 kg ± 22.8 kg. Eighty per cent of the population was overweight (body mass index [BMI] ≥ 25 kg/m^2^), although the majority (65.7%) had an arm circumference smaller than 35 cm. Blood pressure readings were measured using both ABPM and office monitoring techniques. The mean office reading at 141.9/86.1 mm Hg was slightly higher than the mean 24 h ABPM value of 137.9/80 mm Hg. The average ABPM daytime reading was 140.5/82.4 mm Hg.

### Acceptance of ambulatory blood pressure monitoring

The mean Likert scores for acceptance questions are presented in Table 2. Results indicate that the comfort of the monitor was rated favourably (1.79 ± 0.5) by the majority of the participants (82.9%), with the highest score achieved for the monitor’s light weight (1.90 ± 0.3). A small proportion of the population reported experiencing difficulties in utilising the arm where the cuff was attached (1.83 ± 0.4); however, the majority of the participants (90%) were able to execute their daily activities without much discomfort both at home and at work. The extent to which the noise made by the monitor was bothersome was minimal, with only 11% of the participants reporting that it disturbed them whilst at home, and 9% whilst at work. A small proportion (20%) reported that the monitor disturbed other people; however, the majority (68%) did not personally find the monitor embarrassing to wear (1.89 ± 0.4. Sleep interference was a major cause of dissatisfaction (0.61 ± 0.8), with over half of the participants (57%) rating it as a cause of discomfort.

**TABLE 2:** Likert score for acceptance questions.

Acceptance questions	Mean (SD)		Likert score†					
			0		1		2	
	*n*	%	*n*	%	*n*	%	*n*	%
The monitor was comfortable to wear	1.79	0.5	3	4.3	9	12.9	58	82.9
The monitor was light	1.90	0.3	1	1.4	5	7.1	64	91.4
I am able to use my arm while wearing the pressure cuff	1.83	0.4	1	1.4	10	14.3	59	84.3
I managed to do some everyday activities while wearing the monitor at home and at work	1.83	0.4	2	2.9	8	11.4	60	85.7
I managed to do some everyday activities while wearing the monitor at other times (e.g. shopping, socialising, church)	1.87	0.4	2	2.9	5	7.1	63	90
Cuff inflation did not disturb me at home and at work	1.36	0.9	2	2.9	8	11.4	60	85.7
Cuff inflation did not disturb me at other times (e.g. shopping, church, socialising)	1.47	0.8	2	2.9	5	7.1	63	90
The noise of the pump did not disturb me at home and at work	1.80	0.6	6	8.6	2	2.9	62	88.6
The noise of the pump did not disturb me at other times (e.g. shopping, church, socialising)	1.86	0.5	4	5.7	2	2.9	64	91.4
The noise of the pump did not disturb others	1.67	0.7	9	12.9	5	7.1	56	80
The monitor was not embarrassing to wear	1.89	0.4	2	2.9	4	5.7	64	91.4
I was not worried about seeing my BP results	1.79	0.6	5	7.1	5	7.1	60	85.7
Wearing the BP cuff was not stressful	1.60	0.7	7	10	14	20	49	70
My normal sleeping pattern was not disturbed during the monitoring	0.61	0.8	40	57.1	17	24.3	13	18.6
The monitor did not disturb me enough to make me remove it before the end of the study during the day	0.91	0.4	13	18.6	0	0	57	81.4

† , Likert response: 0 – disagree, 1 – neutral, 2 – agree.

BP, blood pressure.

The experience of the ABPM process was generally rated as favourable with over 70% of the population achieving an acceptance score classified as acceptable (≥ 23). The mean acceptance score for all 70 participants was 23.1 ± 4.0 (total score = 30). A third of the participants were classified as having poor acceptance (scores less than the 25th percentile [< 23]).

From Table 3, almost all participants responded positively to these questions. They valued the ABPM process (95.7%), as it roused interest in knowing more about personal BP (95.7%) and provided significant motivation to continue with good medicine-taking practice (97.1%). They reported a willingness to wear the monitor again. Most participants (97.1%) believed that other patients with hypertension would be willing to undergo the ABPM process, as they felt that it would improve their knowledge of, and insight into, their condition and could motivate them to continue taking their medication (98.6%).

**TABLE 3:** Positive outcomes of wearing the monitor (non-Likert) (*n* = 70).

Questions exploring positive outcomes	*n*	%
I am willing to wear the monitor again for 24 h	65	92.9
Wearing the monitor has helped me	67	95.7
I liked seeing my BP readings and how they changed during the day and night	67	95.7
Seeing my BP readings motivated me to continue taking my medicine	68	97.1
Other patients with high BP would be willing to wear the monitor for 24 h in order to check their BP	68	97.1
Wearing the monitor and seeing BP results could motivate other patients who have high BP to continue taking their medication	69	98.6

BP, blood pressure.

Table 4 shows some of the reported negative outcomes of wearing the monitor. Sleep interference presented a major inconvenience of wearing the monitor, with only a minority (25.7%) reporting experiencing no sleep disturbance at any point in the night. Just below a fifth of the participants (17.1%) reported that night readings hindered them from falling asleep, while difficulty maintaining sleep because of the monitor was reported in the majority (74.3%) of the population.

**TABLE 4:** Negative outcomes and adverse effects of wearing the monitor (*n* = 70).

Questions exploring negative outcomes	*n*	%
The monitor prevented me from falling asleep	12	17.1
The monitor woke me after I had fallen asleep	52	74.3
**The number of times woken up by the monitor†**		
0	18	26.7
1	9	12.9
2	11	15.7
≥ 3	32	45.7
Removed the monitor at some point during the study	13	18.6
**Removed monitor**		
During the day	3	4.3
At night	10	14.3
**Adverse effects**		
Pain	13	19.0
Skin irritation	11	16.0
Bruising	–	-

† , Results include participants who removed the monitor at some point during the night.

Extreme sleep hindrance (> 3 awakenings) was reported in 32/70 of the participants; however, the extent to which the monitor was bothersome enough to result in removal was minimal, with only 14.3% reporting that they felt that the monitor disturbed them enough to make them remove it at some point during the night. Reported adverse effects were skin irritation (16%) and pain (19%). No bruising was reported in this population.

### Influence of variables on acceptance of ambulatory blood pressure monitoring

Most socio-demographic characteristics showed no association with acceptance of ABPM; gender (*p* = 0.599), age (*p* = 0.437), health literacy (*p* = 0.170) and employment (*p* = 0.696). Years of schooling was the only socio-demographic variable that showed a weak but significant correlation with the ABPM acceptance score (*r* = -0.243, *p* = 0.042).

No significant relationships were found between acceptance and clinical variables such as BMI (*p* = 0.771), arm circumference (*p* = 0.882), number of comorbidities (*p* = 0.090), medicine regimen (*p* = 0.473) and BP category (*p* = 0.713). Associations between acceptance and adverse effects of wearing the monitor were investigated, but no significant relationship with acceptance was found for either pain (*p* = 0.353) or skin irritation (*p* = 0.473).

The acceptance of the technique was significantly affected by removal of the monitor (*p* = 0.002). Of the 26% of the population that was categorised as having poor acceptance of the technique, 43.9% removed the monitor during the night. Adverse effects such as pain and skin irritation were not associated with poor acceptance, with the majority of individuals with poor acceptance (83%) having experienced no adverse effects.

## Discussion

This study is, to our knowledge, the first to explore the acceptance of the ABPM technique in an African population. The findings of our study, conducted in a primary care setting, revealed that the technique was considered acceptable by 70% of the study population. Participants reported that the ABPM process, which included interacting with the researcher, improved their insight into hypertension and motivated adherence to treatment, in spite of some of the discomforts experienced.

Participants generally considered the experience to have been worthwhile, a finding similar to that of Ernst and Bergus (2003). Most participants declared a willingness to have the 24-h ABPM repeated if advised to do so, supporting similar findings from previous studies (Elliot & Iqbal 2003; Mallion et al. 1996). However, a study in the Netherlands, which compared ABPM acceptance with acceptance of other diagnostic procedures such as home BP monitoring and office BP monitoring, concluded that ABPM had the lowest patient acceptance (Beltman et al. 1996).

In our study, issues of discomfort whilst wearing the monitor were reported by a minority of participants, and most appeared tolerant of monitor noise, similar to the finding of a study in pregnant women who reported the monitor as being reasonably comfortable, and other inconveniences as tolerable (Walker et al. 2004). However, our participants did report some discomfort and inconvenience, with sleep interference being a major cause of dissatisfaction. Over half of the population reported three or more awakenings during the night, but in spite of this, only 14% removed the Ambulatory blood pressure monitor during the night. A number of other similar studies reported sleep interference as a major inconvenience, but these studies are not directly comparable to the present study because of differences in population demographics, sample size and study design (Beltman et al. 1996; Ernst & Bergus 2003; Mallion et al. 1996; Viera et al. 2011; Walker et al. 2004).

As has been observed from other studies dealing with the acceptance of the ABPM technique (Beltman et al. 1996; Mallion et al. 1996; Viera et al. 2011; Walker et al. 2004), pain and skin irritation were the most commonly reported adverse effects in our study. Whilst skin bruising was not a reported adverse effect in our study population, others have reported its occurrence, albeit less frequently than other adverse effects (Ernst & Bergus 2003; Viera et al. 2011). Participants generally considered the experience to have been worthwhile, with most being willing to have 24-h ABPM repeated if advised to do so, supporting previous findings (Elliot & Iqbal 2003; Ernst & Bergus 2003; Mallion et al. 1996).

Acceptance of health interventions and medical technologies has been associated with factors such as socio-demographic characteristics, beliefs and perceived usefulness of the technique (Calnan et al. 2005; Peek et al. 2014). Our findings indicated that acceptance was influenced only by education, with those who reported more years of schooling having better acceptance of the technique, a finding supported by others (Ernst & Bergus 2003). Participants with more years of schooling may have better appreciated the benefits of the ABPM process and were therefore more willing to accommodate any associated inconveniences.

Our health literacy assessment showed that almost three quarters of our participants had inadequate health literacy, in spite of using a health literacy measure that was not cognitively demanding. We had postulated a relatively low acceptance of the technique in our population, as limited literacy is linked to decreased understanding of medical advice as well as difficulties in understanding the importance and utilisation of health services, (Friis et al. 2016; Nutbeam 2000; Weiss et al. 1992; Williams et al. 1995). Interestingly however, the findings of our study in a population from an LMIC were relatively consistent with those from high-income countries, in spite of major differences in the health care systems, culture, health-related beliefs and health literacy skills. We found no significant associations between ABPM acceptance and gender, age, height, weight, BMI, arm circumference, office BP, 24-h ABPM profile or adverse effects or inconveniences of wearing the monitor. However, a unique, previously unreported finding was that our participants reported that the process stimulated an interest in knowing more about personal BP, providing significant motivation to continue with good medicine-taking practice, and to actively engage in self-care.

## Limitations

The study was limited by a small sample size that could have affected attempts to establish statistically significant relationships. Participants were drawn from five clinics in the same town in one province of South Africa, limiting generalisability of the findings, as this population group is not necessarily representative of all ethnic and economic groups within South Africa. The majority of the participants in this study spoke isiXhosa as their first language, whereas the researcher did not speak isiXhosa. This presented both language and cultural barriers and may have resulted in some details being omitted or falsely interpreted, thereby affecting the accuracy of the results produced. The employment of an isiXhosa-speaking interpreter assisted with language translation in all interviews. A strength of the study was that all data were collected by one researcher (F.C.).

## Implications and recommendations

Our research has shown that the majority of the participants found the ABPM technique to be acceptable and appreciated the value of the information provided by the ABPM process. This implies that the routine utilisation of the ABPM technique at the PHC level could be implemented in this population. As the socio-demographic and socio-economic characteristics of the South African public- and private-sector populations are very different, further research could include comparative studies in these two patient populations in a multicentre study to compare acceptance and the determinants influencing acceptance of this technique.

Of particular interest is the motivational power or influence of the ABPM process, which reportedly stimulated a desire in patients to learn more about their BP and about hypertension in general. Undergoing the ABPM process may encourage hypertensive patients to engage in self-management practices. This might help in effectively controlling hypertension particularly in the current healthcare system, where there are shortages in healthcare professionals, resulting in limited healthcare professional–patient interaction. In this light, it is postulated that findings from the current study are vitally important, given the notably high prevalence of hypertension in South Africa and can therefore be used to inform current practice and future research on the subject matter.

## Conclusion

In a first of its kind in Africa, this study found that a large proportion of the participants showed acceptance of the ABPM technique, in spite of reported inconveniences. Sleep interference, pain and skin irritation were the major reported causes of dissatisfaction, although most participants were still able to continue wearing the cuff for the 24 h monitoring period. Additional value offered by the ABPM process was the insight it afforded into individual results, stimulating a desire to learn more about their condition. Given the high prevalence of hypertension in South Africa and the local acceptance of this technique, ABPM could be a valuable tool in both practice, and also serve as a stimulus for further education of patients with hypertension.
